# Dynamic Support Culture of Murine Skeletal Muscle-Derived Stem Cells Improves Their Cardiogenic Potential *In Vitro*

**DOI:** 10.1155/2015/247091

**Published:** 2015-08-18

**Authors:** Klaus Neef, Philipp Treskes, Guoxing Xu, Florian Drey, Sureshkumar Perumal Srinivasan, Tomo Saric, Erastus Nembo, Judith Semmler, Filomain Nguemo, Christof Stamm, Douglas B. Cowan, Antje-Christin Deppe, Maximilian Scherner, Thorsten Wittwer, Jürgen Hescheler, Thorsten Wahlers, Yeong-Hoon Choi

**Affiliations:** ^1^Department of Cardiothoracic Surgery, Heart Center, University of Cologne, 50937 Cologne, Germany; ^2^Center for Molecular Medicine Cologne, University of Cologne, 50931 Cologne, Germany; ^3^Institute for Neurophysiology, University of Cologne, 50931 Cologne, Germany; ^4^Berlin-Brandenburg Center for Regenerative Therapies, 13353 Berlin, Germany; ^5^Department of Anesthesiology, Perioperative and Pain Medicine, Children's Hospital Boston and Harvard Medical School, Boston, MA 02115, USA

## Abstract

Ischemic heart disease is the main cause of death in western countries and its burden is increasing worldwide. It typically involves irreversible degeneration and loss of myocardial tissue leading to poor prognosis and fatal outcome. Autologous cells with the potential to regenerate damaged heart tissue would be an ideal source for cell therapeutic approaches. Here, we compared different methods of conditional culture for increasing the yield and cardiogenic potential of murine skeletal muscle-derived stem cells. A subpopulation of nonadherent cells was isolated from skeletal muscle by preplating and applying cell culture conditions differing in support of cluster formation. In contrast to static culture conditions, dynamic culture with or without previous hanging drop preculture led to significantly increased cluster diameters and the expression of cardiac specific markers on the protein and mRNA level. Whole-cell patch-clamp studies revealed similarities to pacemaker action potentials and responsiveness to cardiac specific pharmacological stimuli. This data indicates that skeletal muscle-derived stem cells are capable of adopting enhanced cardiac muscle cell-like properties by applying specific culture conditions. Choosing this route for the establishment of a sustainable, autologous source of cells for cardiac therapies holds the potential of being clinically more acceptable than transgenic manipulation of cells.

## 1. Introduction

Ischemic heart disease is the most common cause of death worldwide [1] and is characterized by degeneration of heart muscle tissue as a consequence of cell death resulting from shortage of oxygen and nutritional supply. Typically, this will result in cardiac insufficiency and ultimately heart failure, causing substantial socioeconomic burden, most prominent in developed countries, but increasingly throughout the world. The human left ventricle contains approximately 2 to 4 × 10^9^ cardiomyocytes (CMs), of which as much as 25% can be lost in a single nonfatal event of myocardial infarction (MI) [2]. Since the adult mammalian myocardium has only very limited potential to regenerate [3], research on cardiac cell therapy aims at developing methods to repair damaged heart tissue by transplantation of therapeutically effective cells [4, 5].

Various cell types have been tested for efficacy in cardiac cell therapy in animal models and early clinical settings. Since the most obvious choice of cells, functional CMs, are not available in relevant numbers due to their limited proliferation potential* in vitro*, alternative cell populations, mostly stem or progenitor cells, have been investigated. Early studies concentrated on bone marrow-derived cells, due to their relative ease of acquisition from bone marrow aspirates and established regenerative potential for hematopoiesis and angiogenesis [6]. General safety and moderate therapeutic efficacy of these cells for treatment of acute cardiac infarction have been shown in a meta-analysis of clinical trials [7]. Recently, promising results from clinical studies using cardiac stem cells derived from patient myocardial tissue have been published [8], but the underlying biological mechanisms remain unresolved [9].

Another autologous cell source which has been used in the context of cardiac cell therapy is skeletal muscle progenitor cells, which are recruited from satellite cells in response to muscle injury* in situ* and proliferate as skeletal myoblasts (MBs)* in vitro* [10]. Here, despite initially promising results in animal models [11, 12] and clinical trials [13, 14], safety issues became apparent after arrhythmias had been observed in patients receiving MBs after myocardial infarction [13, 15], most likely due to electrophysiological isolation of transplanted cells [16, 17]. Consequently, when considering MBs as an option for cardiac cell therapy, prior modification of cells is advisable, as shown recently by our group using a nontransgenic approach [18] or by transplantation of transgenic MBs expressing cardiac gap junction proteins [19].

A variety of publications have reported that skeletal muscle additionally harbors a subpopulation of multipotent stem cells, which have been termed muscle-derived stem cells (MDSCs) and are subject to controversial discussion [20–23]. To utilize the full potential of MDSCs as a source of autologous cells for cardiac cell therapy, further clarification of their cellular identity, differentiation potential, functional properties, and therapeutic efficacy is required. During the isolation of MDSCs from muscle tissue a consistently reported characteristic feature, often used for separation from MBs and fibroblasts [18], is a propensity for nonadherence to cell culture plastic surfaces and the formation of cell clusters.

Our aim was to exploit this feature by supporting nonadherence and cluster formation in early isolations of MDSCs via the application of specific culture conditions. By observing cell morphology, together with expression and functional electrophysiological studies, we could confirm an improved cardiogenic potential of these MDSCs in response to dynamic support culture compared to standard culture* in vitro*.

## 2. Materials and Methods

### 2.1. Tissue Processing and Cell Isolation

Cells were isolated from skeletal muscles of forelimbs and hindlimbs of neonatal C57BL/6 mice as previously described [18, 24]. Briefly, muscle tissue was minced and freed from connective tissue residue by enzymatic digestion in phosphate buffer saline (PBS; Invitrogen, Karlsruhe, Germany), containing 0.2% collagenase type IV and 2.4 IU/mL dispase (Invitrogen) and 3 mM calcium chloride (Sigma-Aldrich, Munich, Germany). Primary cell isolates were filtered using a 70 *μ*m cell strainer (BD Biosciences, Heidelberg, Germany) and cultured in DMEM/F12 medium with 5% fetal bovine serum, 1% ITS-X, 1% Penicillin/Streptomycin, 0.5 *μ*g/mL Fungizone (all Invitrogen, Karlsruhe, Germany), 10 ng/mL recombinant human basic fibroblast growth factor, and 10 ng/mL recombinant human epidermal growth factor (both PeproTech, Hamburg, Germany). These cells were subjected to serial preplating steps 2 h (pP1), 26 h (pP2), and 74 h (pP3) after isolation in 10 cm cell culture dishes (Falcon, BD, Heidelberg, Germany). After each preplating step only nonadherent cells were passaged, while adherent cells were discarded. After pP3, nonadherent cells were defined as day 0 cells (*ISH0*) and cultured (10^5^ cells/cm²) using three different cell culture conditions:* I* (incubator), referring to the incubation of cells applying static conditions in a standard cell culture incubator at 37°C and 5% CO_2_;* S* (shaker), referring to incubation on a horizontal rocking platform at 50 rpm;* H* (hanging drop), referring to initial incubation for 48 h in hanging drops (6 × 10^4^ cells/20 *μ*L drop) at 37°C and 5% CO_2_, followed by S culture conditions.

At days 4, 8, and 12, nonadherent cells were collected, counted, and passaged. Populations of nonadherent cells were termed according to the collection day and the condition applied, that is, H12: hanging drop condition at day 12. Cluster diameters were measured from microscopic images (2.5x magnification,* Axiovert 25*, Zeiss, Oberkochen, Germany). A minimum of 3 images from samples (ISH0, I12, S12, and H12) of each isolation (*n* = 5) were analyzed using* AxioVision* 4.5 software (Zeiss). Cell numbers were assessed from samples acquired during passaging. Samples were incubated with Accutase (Invitrogen) for 15 minutes at 37°C to dissociate clusters. Cells were counted using a* Neubauer* hemocytometer (Marienfeld, Lauda-Königshofen, Germany). MBs [18] and embryonic stem cell (ESC) derived CMs [25] were used as controls for immunocytochemistry and quantitative real-time PCR (qPCR).

### 2.2. Immunocytochemistry

For immunocytochemical staining, either intact or Accutase dissociated clusters were centrifuged (500 g, 10 minutes) onto fibronectin coated (2.5 *μ*g/mL; Sigma-Aldrich, Taufkirchen, Germany) coverslips and further incubated for 72 h before analysis. The samples were fixed with 4% paraformaldehyde, permeabilized with 0.25% Triton X-100/0.5 M NH_4_Cl, and blocked with 5% goat serum (all Sigma-Aldrich) in PBS (Invitrogen). Samples were stained with 4,6-diamidino-2-phenylindole (DAP; Invitrogen). Primary and secondary antibodies (see Table S1 in the Supplementary Material available online at http://dx.doi.org/10.1155/2015/247091) were diluted in PBS with 1% bovine serum albumin (BSA, Invitrogen). Fluorescence microscopy was performed using a* Ti-U* microscope and* NIS Elements BR* 3.10 software (both Nikon, Düsseldorf, Germany). Ratios of cells positive for marker expression were assessed by analyzing 5 fields of vision (20x magnification) for 3 biological replicates (i.e., a total of >500 cells were analyzed per marker and sample). Specificity of staining was tested by appropriate controls (Figures S3 to S7).

### 2.3. Flow Cytometry

For flow cytometric analyses of intracellular markers, single cells from Accutase dissociated clusters were fixed and permeabilized with* Cytofix/Cytoperm* solution (BD). PEB (PBS with 0.5% BSA and 2 mM ethylenediaminetetraacetic acid, EDTA, Sigma-Aldrich) was used for dilution of antibodies, washing, and incubation. Table S1 lists detailed information about antibodies used. Measurements were performed on a* FACSCalibur* flow cytometer with* CellQuest Pro 6* software (both BD).

### 2.4. Quantitative Real-Time PCR

After a final static incubation for 72 h, a minimum of 5 × 10^5^ cells from all conditions were used for total RNA extraction using the* Nucleospin RNA XS* kit (Macherey Nagel, Düren, Germany), followed by reverse transcription, using the* High Capacity* cDNA reverse transcription kit and DNase I treatment (both Invitrogen).* SYBR Green Power Mix*, 10 mM oligonucleotide primers (both Invitrogen), and 10 ng cDNA per reaction were used for quantification of gene expression on a* StepOne Plus* real-time PCR system (Applied Biosystems, ABI, Darmstadt, Germany). Data analysis was performed using* StepOne 2.2* software (ABI). See Table S2 for primer sequences.

### 2.5. Patch-Clamp Analysis

Electrophysiological properties of MDSCs were assessed using whole-cell patch-clamping [26]. Cells from dissociated clusters were plated on fibronectin coated coverslips as described for immunocytochemistry. Pipettes (3–5 MΩ resistance when filled with standard intracellular solution, Table S3A) were made from thin walled borosilicate glass capillaries tubes (World Precision Instruments WPI) on a Zeitz DMZ Universal Puller (DMZ). All recordings were performed one or two days after cell plating, using an* EPC 9* amplifier with* Pulse* software (HEKA Instruments, Lambrecht, Germany), applying continuous perfusion with buffer (extracellular solution, Table S3B) The bath temperature was held constant at 37°C. After establishment of the gigaohmic seal, membrane capacitance *C*_*m*_ and series resistance *R*_series_ were compensated to minimize the capacitive transient. Only cells showing stable values were included in the analysis. APs were recorded in current-clamp mode and funny current (*I*_*f*_) in voltage clamp mode. For *I*_*f*_ recording, hyperpolarizing steps from a holding potential of −40 mV to the range of −150 to −100 mV in 10 mV-steps were applied. Data were digitized at 10 kHz, filtered at 1 kHz, and stored on hard disk. Beating frequency was measured as the number of APs per minute over the duration of 5 minutes. For indicated measurements, 0.5 mM CdCl_2_ or 1 *μ*M isoproterenol (both Sigma-Aldrich) was added to the buffer.

### 2.6. Statistical Analysis

Statistical analysis was performed using the* SigmaStat* 4 software (Systat Software GmbH, Erkrath, Germany). Comparison of groups was made using a one-way analysis of variance (ANOVA) followed by a* post hoc* Bonferroni test for multigroup comparisons or via Student's *t*-test for single group comparisons (*p* < 0.05 considered significant). Data is shown as mean ± standard error of the mean (SEM) unless stated otherwise.

## 3. Results

### 3.1. Skeletal Muscle Preparation and Initial Cell Characterization

**Figure 1:**
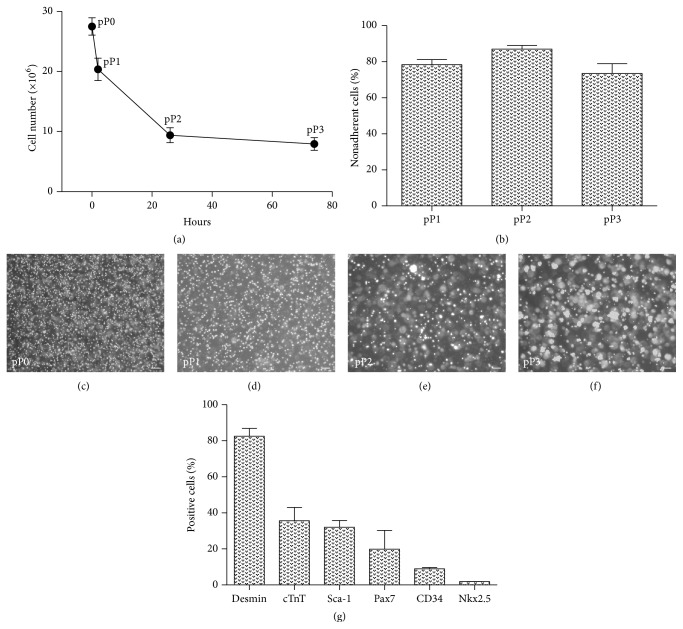
Isolation of MDSCs from neonatal murine skeletal muscles. (a) Total number of nonadherent cells (per g of muscle tissue) and ratios of nonadherent cells (b) during three preplating steps (pP1–pP3). Panels (c–f) show representative images of nonadherent cells during the preplating procedure: before plating (pP0, (c)), 2 hours (pP1, (d)), 26 hours (pP2, (e)), and 74 hours (pP3, (f)) after plating. Scale: 100 *μ*m. (g) Flow cytometric assessment of cardiac and skeletal muscle specific markers for cells from ISH0 cell population (*n* = 5).

The mechanical and enzymatic dissociation of skeletal muscles isolated from neonatal mice resulted in 27.5 ± 1.4 × 10^6^ per gram of tissue (*n* = 12). Three serial preplating steps (pP1–pP3) reduced cell numbers, that is, numbers of vital nonadherent cells, to 20.3 ± 1.9 × 10^6^ after pP1, to 9.4 ± 1.2 × 10^6^ after pP2, and to 7.9 ± 1.0 × 10^6^ after pP3 (Figure 1). The resulting population of nonadherent, cluster-forming cells after pP3 was termed ISH0, since it served as the initial population (day 0) of cells, which was then split and subjected to three different cell culture conditions: static incubation (*I*), dynamic incubation on a horizontal shaker (*S*), and preculture in hanging drops (*H*) with subsequent culture on a shaker. ISH0 cells formed clusters of spontaneously beating cells. Flow cytometric analyses (*n* = 5) revealed a heterogeneous cell population with a majority of cells expressing the pan-muscle marker desmin (82.5%  ± 4.4%) and substantial fractions of cells expressing cardiac troponin T (cTnT, 35.6%  ± 7.4%), stem cell lineage marker Sca-1 (32.0%  ± 3.7%), and skeletal muscle progenitor cell specific transcription factor Pax7 (19.9%  ± 10.4%). Additionally, smaller fractions of cells were also positive for the hematopoietic stem cell marker CD34 (9.0%  ± 0.8%) and cardiac transcription factor Nkx2.5 (1.9%  ± 0.1%).

### 3.2. Impact of Dynamic Support Culture on Cluster Morphology and Cell Numbers

**Figure 2:**
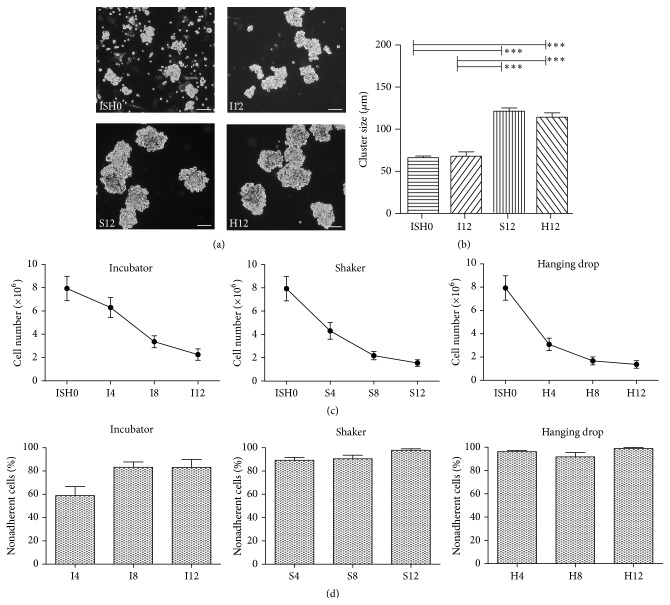
Characteristics of cell clusters derived from MDSCs. (a) Representative phase contrast microphotographs of cell clusters from different cell culture conditions. While single contracting cells and smaller clusters were visible in ISH0 cultures, most cells were organized in clusters of loosely attached cells applying static (I) and dynamic (S and H) culture conditions at day 12 of cultivation (I12, S12, and H12). Scale: 100 *μ*m. (b) Average sizes of clusters in initial ISH0 cell populations and after twelve days applying different culture conditions. ^*∗∗∗*^ *p* < 0.001. (c) Total numbers of nonadherent cells obtained from muscles of 10 neonatal mice over the course of 12 days applying different culture conditions (*n* = 12). (d) Ratios of nonadherent cells over the course of 12 days applying different culture conditions (*n* = 12).

Sizes of cell clusters (Figures 2(a) and 2(b)) after 12 days of static culture conditions did not differ significantly from initial ISH0 cell clusters (ISH0: 66.4 ± 2.0 *μ*m, *n* = 9; I12: 68.2 ± 5.2 *μ*m, *n* = 5), but clusters were significantly larger under both dynamic conditions (S12: 121.2 ± 3.9 *μ*m; H12: 114.7 ± 5.2 *μ*m; both *p* < 0.001 versus ISH0 and I12). Development of the number of nonadherent cells counted in suspension over the course of 12 days did not differ significantly among I, S, and H and showed a loss of approximately 6% of nonadherent cells per day, resulting in 28.4% (I: 3.13 ± 2.36 × 10^6^ cells), 19.4% (S: 2.14 ± 1.34 × 10^6^ cells), and 17.3% (H: 1.9 ± 1.53 × 10^6^ cells) of the original ISH0 cells remaining after 12 days (Figure 2(c)). Although cell numbers continually declined during 12 days of static or dynamic culture (Figure 2(c)) an increase in ratios of nonadherent cells was observed (Figure 2(d)).

### 3.3. Impact of Dynamic Support Culture on Cardiac Marker Expression

**Figure 3:**
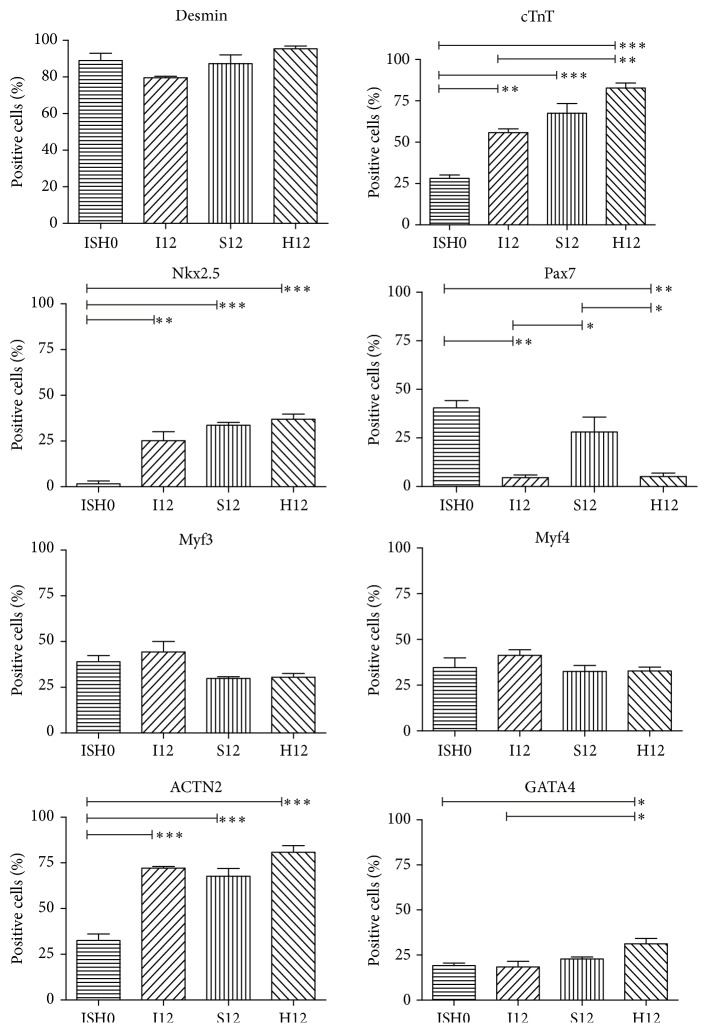
Immunocytochemical quantification of skeletal and cardiac muscle markers in MDSCs cultured under dynamic support. Cell clusters from the initial cell population (ISH0) and after applying different cell culture conditions for 12 days (I12, S12, and H12) were dissociated, plated, and stained for desmin, cTnT, Nkx2.5, Pax7, Myf3, Myf4, ACTN2, and Gata4. Ratios were calculated as positive cells (stained for marker) versus total cells (stained for DAPI). ^*∗*/*∗∗*/*∗∗∗*^ *p* < 0.05/0.01/0.001.

Clusters from ISH0 and after 12 days of cultivation under different conditions were analyzed immunocytochemically for the expression of cardiac and myogenic markers. Staining of intact clusters did not reveal marker expression to be localized to specific regions of a cluster (Figures S1 and S2). Thus, immunocytochemical analyses were performed on single cells from dissociated clusters. Specificity of antibody staining was confirmed with appropriate controls (Figure S3). Quantification of ratios of cells expressing skeletal and cardiac muscle markers desmin, cTnT, Pax7, and Nkx2.5 in ISH0 confirmed the results acquired by flow cytometric analysis (Figures 3 and S4). Further analyses covered myogenic regulatory factors 3 and 4 (Myf3 and Myf4), cardiac specific *α*-actinin 2 (ACTN2), and cardiac specific transcription factor GATA4 and revealed ratios of >35% Myf3 and Myf4 positive cells and >80% desmin positive cells for ISH0 and all three culture conditions at day 12 (Figures 3 and S4–S7). The ratios of cells expressing Pax7 decreased significantly until day 12 for all culture conditions except S12, while ratios of cells that expressed the cardiac markers ACTN2, cTnT, and Nkx2.5 increased significantly after ISH0 for all conditions. Compared to ISH0, the expression of GATA4 was similar in I12 and S12 but was significantly increased in H12 (Figures 3 and S4–S7).

**Figure 4:**
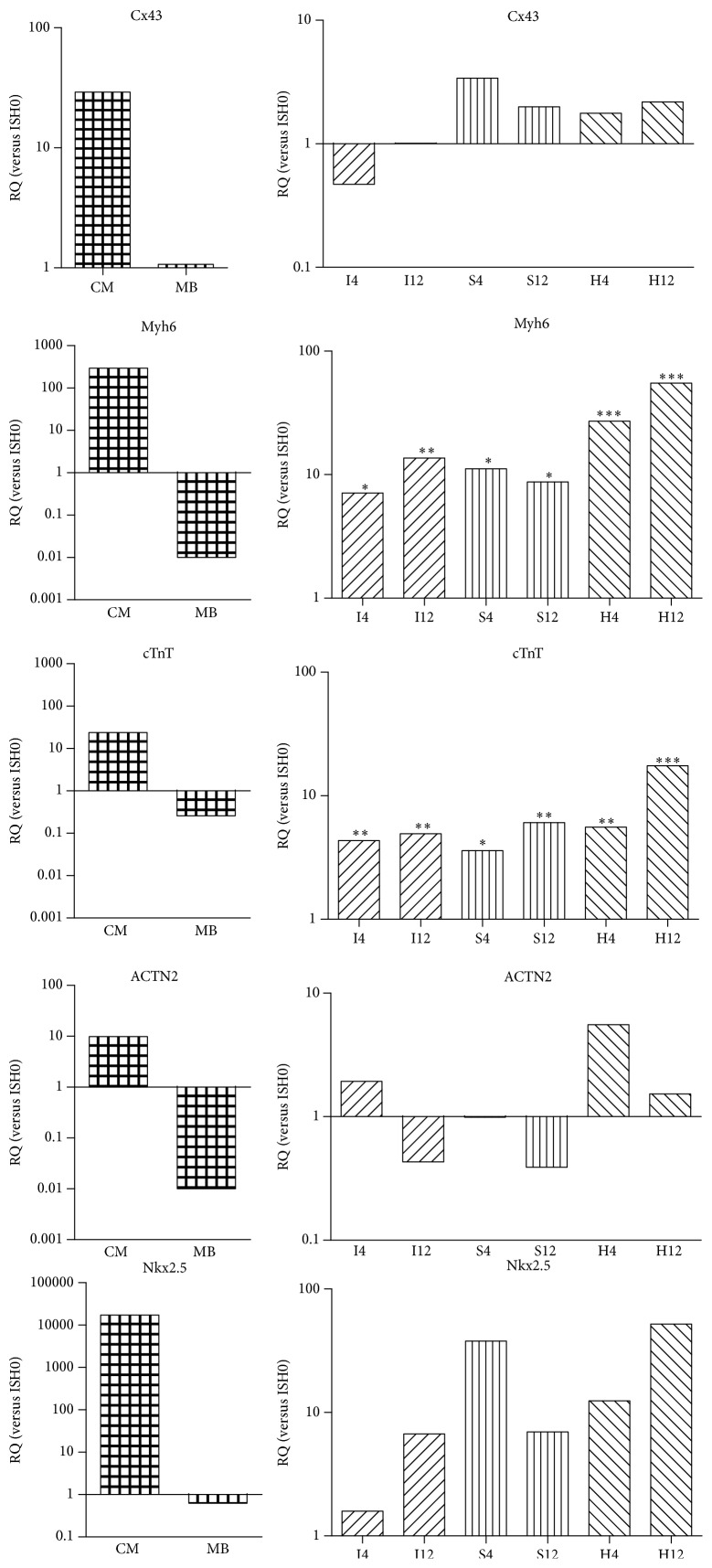
Expression of cardiomyocyte-specific transcripts in MDSCs cultured under dynamic support. Skeletal myoblasts, purified embryonic stem cell-derived cardiomyocytes, and cells from I, S, and H cell culture conditions on days 4 and 12 of cultivation were analyzed by qPCR. Expression levels of indicated genes were normalized to the reference gene *β*-actin and displayed as relative expression compared to ISH0 cells. Calculation of statistical significance was based on ΔCt values (see Figure S8). ^*∗*/*∗∗*/*∗∗∗*^ *p* < 0.05/0.01/0.001.

The expression of connexin 43 (Cx43), *α*-myosin heavy chain 6 (MYH6), cTnT, ACTN2, and Nkx2.5 were analyzed by qPCR on the transcript level. Purified ESC derived CMs and MBs were used as controls. Compared to CMs and MBs, ISH0 showed an intermediate expression level for MYH6, cTnT, and Nkx2.5, while the expression level for Cx43 of ISH0 was similar to MBs (Figures 4 and S8). Comparing cells from day 4 and day 12 of the three different culture conditions to ISH0 cells, the expressions of MYH6 and cTnT were already increased more than 5-fold in cells at day 4 (I4 and S4 versus ISH0: *p* < 0.05; H4 versus ISH0: *p* < 0.001) and further increased in I12 (13.6-fold; *p* < 0.01 versus ISH0) and H12 cells (55.2-fold; *p* < 0.001 versus ISH0). The expression levels of cTnT were more than 3-fold higher in cells at day 4 in all cell culture conditions compared to ISH0 (I4 and H4 versus ISH0: *p* < 0.01; S4 versus ISH0: *p* < 0.05). The highest level of cTnT expression was detected in H12 cells (17.5-fold; *p* < 0.001 versus ISH0). During 12-day cultivation in all three culture conditions, Cx43 and ACTN2 showed only minor changes in expression levels, ranging from 0.5- to 2-fold (Figures 4 and S8). Nkx2.5 showed a tendency for increased expression, especially for condition H (H4: 27x, H12: 182x), but without reaching significance, presumably, because of very low overall expression levels (1,000-fold less than endogenous control).

### 3.4. Functional Characteristics of MDSCs Cultured under Dynamic Support* In Vitro*

The observation, that cells in clusters of MDSCs cultured under dynamic support express cardiac specific proteins and transcripts, prompted us to closer explore their functional properties. For this reason electrophysiological patch-clamp measurements were performed on spontaneously beating single cells obtained from ISH0 clusters and day 12 samples applying different conditions. Spontaneous contractions of cells from ISH0 occurred irregularly and frequently stopped after a seal was established. Out of 51 cells that were patch-clamped, 3 cells (5.9%) could be successfully analyzed, all of which exhibited irregular beating, characterized by short episodes of burst-like activity. After 12 days of cultivation, spontaneous cell contractions were more stable and regular in each culture condition: in the I12 group, out of 77 analyzed, 8 (10.4%) cells showed regular and 7 (9.1%) irregular beating activity and in the S12 group, 37 cells were patched and 8 (21.6%) showed regular and 4 (10.8%) irregular beating, while in the H12 group, out of 89 measured cells, 9 (10.1%) exhibited regular and 5 (5.6%) cells irregular activity. Representative traces of irregularly and regularly beating MDSCs are displayed in Figure S9.

**Figure 5:**
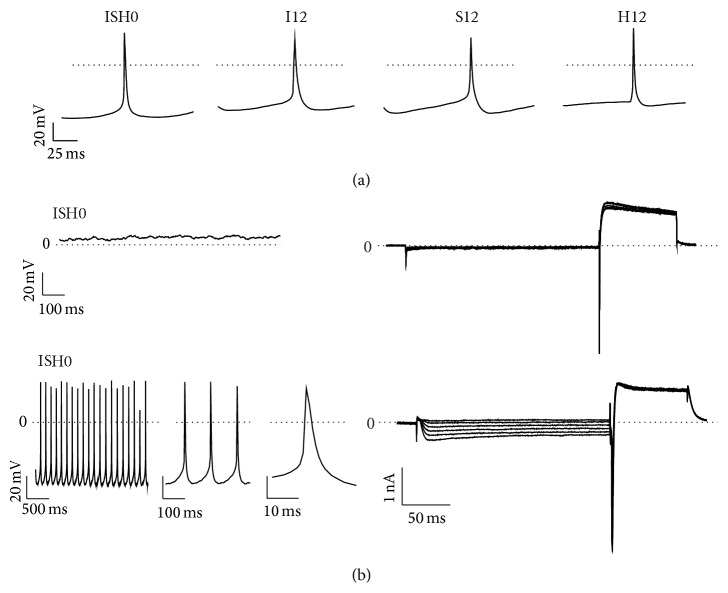
Electrophysiological analyses of MDSC-derived cells. (a) Representative action potential traces of initial cell population (ISH0) and after applying different cell culture conditions for 12 days (I12, S12, and H12) as measured by a whole-cell patch-clamp in current-clamp mode. (b) Voltage clamp measurements for recording of the pacemaker current *I*_*f*_. The applied voltage protocol is shown above the traces, which are representative for cells which do not express (upper trace) and which express small (lower trace) *I*_*f*_ currents.

The frequency of spontaneous action potentials (APs) was similar in ISH0 (360.6 ± 81.6 beats/min) and H12 cells (444.4 ± 39.3 beats/min), while cells from I12 and S12 showed more than 2x higher frequencies (Table 1). Analysis of AP parameters revealed that I12 cells showed a more depolarized maximum diastolic potential (MDP), shorter AP duration (APD), and lower maximum AP upstroke velocity (*V*_max_) than ISH0, S12, and H12 cells (Figure 5(b)). However, the morphology of APs from cells cultured under different conditions was similar, characterized by a slow depolarization before each AP, fast upstroke, and short AP duration, without a plateau phase after the AP upstroke (Figure 5(a)). Further, APs (Figure 5(b),* left panel*) and *I*_*f*_ currents (Figure 5(b),* right panel*) were recorded from the same cell by switching from current-clamping to voltage-clamping as previously described [27]. We examined the functional expression of *I*_*f*_ in spontaneous beating cells generated under ISH10, I12, S12, and H12. The typical representative *I*_*f*_ current traces (Figure 5(b), right) recorded on nonbeating (*upper*) and beating (*lower*) ISH10 cells are depicted. Several beating cells revealed the presence of *I*_*f*_ current whereas most of the nonbeating cells were characterized by the absence of *I*_*f*_ current, confirming the important role of *I*_*f*_ in the generation and modulation of spontaneous beating activity of the cells.

We further determined whether ISH0, I12, S12, and H12 cells responded to cardiac channel specific chemical or pharmacological stimuli (representative traces are displayed in Figure S9). Application of 0.5 mM CdCl_2_, which selectively blocks cardiac AP generation, but has no effect on skeletal myotubes [21], abolished spontaneous beating of cells in all groups (ISH0: *n* = 1, I12: *n* = 5, S12: *n* = 2, H12: *n* = 4). Exposing the cells to *β*1-adrenergic agonist isoproterenol (1 *μ*M) increased the AP frequencies in cells from all culture conditions (ISH0: *n* = 1, I12: *n* = 1, S12: *n* = 2, H12: *n* = 2). Both effects were reversible by washout.

## 4. Discussion

In this study, we sought to investigate whether cells isolated from skeletal muscle can be altered in a nontransgenic way to acquire CM-like properties, eventually allowing functional integration into damaged myocardium to improve heart function. In order to achieve the transition of skeletal muscle-derived cells to CM-like cells various approaches have been followed. Since skeletal muscle cells generally have a contractile and electrically excitable phenotype, changes required for adoption of a cardiac phenotype might be less drastic than, for example, transgenic conversion of fibroblasts to CMs [28]. The isolation of neonatal skeletal muscle cells with CM-like features or cardiogenic potential has been described before [21], but information regarding the influence of specific culture conditions is rare. The choice of neonatal skeletal muscle tissue as a source of potentially cardiogenic cell population has been shown to be instrumental, since adult skeletal muscle tissue yields only marginal amounts of cells viable in cell culture.

The initial nonadherent cell population (ISH0) obtained after skeletal muscle dissociation and preplating was phenotypically heterogeneous, as shown by flow cytometric analysis. The great majority of cells expressed the pan-muscle marker desmin and about 30% were positive for stem cell marker Sca-1, indicative of MDSCs [20]. The cardiac structural protein cTnT was expressed at similar levels, suggesting cardiogenic potential already in the initial cell population ISH0.

Methods that support clustering of cells, like the hanging drop technique [29] and mass suspension cultures [30], have been described to increase cardiac lineage differentiation of pluripotent stem cells and the generation of cardiospheres [31]. We sought to explore if these culture methods could increase the cardiogenic potential of MDSCs and if acceptable cell numbers for subsequent analyses and applications could be generated. As expected, after 12 days of cell culture, clusters obtained applying dynamic conditions (S and H) were significantly larger than those found in conventional static conditions (I), while the total number of cells in clusters remained similar in all three conditions. However, a continuous loss of cells was observed and only about 22% of the initial ISH0 cell population remained after 12 days. A growth lag was described previously for MDSCs and is as such not a surprise [32]. Apparently, however, dynamic culture does not promote cell proliferation over standard incubator culture. Thus, in light of necessary expansion of cells to achieve clinically relevant cell numbers, culture conditions need to be further optimized, for example, by supplementing appropriate growth factors, small molecules, or extracellular matrix components to counter the observed loss of cells or improve proliferation [33].

Analyses of cardiac specific markers confirmed that cells with CM-like expression patterns were already present in the initial ISH0 cell population and showed that these cells could be enriched after 12 days of expansion, applying cluster promoting conditions, as reflected by significantly increased ratios of cells expressing cardiac markers ACTN2, cTnT, and Nkx2.5. Remarkably, expression levels of skeletal muscle markers Myf3 and Myf4 remained constant for all conditions applied. This finding is consistent with a recent report, demonstrating that the expression of these markers is independent of cluster size and that their sustained expression does not influence the cells cardiogenic potential* in vivo* [34], with the general presence of skeletal muscle markers during MDSC isolation [32]. It is important to note that the expression of Pax7 was defined as indicating the presence of skeletal muscle progenitor cells and was not understood as a marker of skeletal myogenic potential but primarily as a reflection of the populations stemness and proliferation potential [35], which apparently remains present to some degree during dynamic support and is in agreement with cluster size correlations in clonal studies [36].

qPCR analyses revealed that cardiac markers were expressed significantly lower in ISH0 cells compared to ESC derived CMs, which were understood as a prototypical stem cell population of high cardiogenic potential in this setup, but were significantly higher expressed than in MBs, which were used as the baseline control for cardiogenic potential of skeletal muscle cells. Comparing the expression levels between the initial ISH0 cells and after 12 days of cell culture revealed that the expression of cardiac structural proteins Myh6 and cTnT was significantly increased in all three conditions. Interestingly, cells from dynamic culture conditions (S and H) showed the highest expression levels, although not reaching the expression levels of ESC derived CMs. This suggests that full transition of MDSCs to a CM-like phenotype* in vitro* requires further optimization. The fact that the expression of late cardiac structural (Myh6 and cTnT) and functional markers (Cx43) is comparable to levels in ESC derived CMs is in our eyes more relevant than the expression of basic sarcomeric structural markers like ACTN2 or early markers of cardiac determination (Nkx2.5) and makes a point for the principal maturation of the cells towards a cardiac phenotype, which is most pronounced under dynamic culture conditions. This increase of cardiac marker expression in dynamic culture was consistent with a previous report in which the higher cell content in clonal clusters was positively correlated with increased cardiac marker expression and cardiac differentiation potential [34].

While, given the difference in source tissue, unsurprisingly not every cardiac marker shows the same relative expression levels in MDSCs cultured under dynamic support as compared to the dedicated high cardiogenic potential population of ESC derived CMs, our analyses make clear that dynamic support culture of MDSCs incrementally narrows the deterministic gap between these cell populations towards a cardiac-like development.

Since MDSCs showed spontaneous beating activity which was shown to be linked to the cardiac specific *I*_*f*_ current [37] and expressed CM-specific markers, especially in cells obtained in dynamic cultures, we analyzed the electrophysiological properties of these cells by whole-cell patch-clamp measurements. Action potentials could be recorded from cells of all conditions, documenting their general electrophysiological competence. The beating frequencies measured (440 to 820 beats/min at day 12) were much higher compared to murine ESC and induced pluripotent stem cell derived CMs, which range between 70 and 160 beats/min [26], thus being more similar to the physiological beating frequency of adult mouse hearts, which reportedly ranges between 450 and 800 beats/min [38]. During 12 days of culture the resting potential and spontaneous beating frequencies of cells cultured applying all conditions increased, while their AP duration decreased, which is consistent with observations of murine pluripotent stem cells differentiating to CMs [26]. Morphological analyses of APs measured in cells of each condition were, however, different from mature adult murine ventricular CM APs [39] and murine ESC derived atrial-like CMs and ventricular-like CM APs [26]. The overall AP morphology was reminiscent of pacemaker cells, which are characterized by a lower MDP and amplitude and a higher frequency than atrial and ventricular APs [40, 41].

It has been shown before that MDSCs react to external chemical stimulation in a manner similar to CMs, characterized by a reversible increase in beating frequency in response to isoproterenol and a reversible decrease in beating frequency in response to CdCl_2_, while skeletal muscle cells and myotubes were not affected [21]. In our hands, these measurements proved to be technically challenging, but cells from all indicated populations, including ISH0, when successfully measured, showed reversible CM-like responses to these substances, indicating CM-like phenotype and functionality, confirming the aforementioned study's findings.

All results considered that cells from the H12 population displayed the most promising combined features of a conversion from a skeletal towards a cardiac phenotype* in vitro*. The correlation between the increased cardiogenic potential and the dynamic support these cells were exposed to is the core finding of our presented study.

## 5. Conclusion

We showed that enforced and sustained nonadherence and cluster formation through dynamic culture and additionally hanging drop pretreatment of MDSCs significantly improved their transition towards a CM-like phenotype. In the future, it will be exciting to tailor the dynamic support culture protocols towards the enrichment of a ventricular CM-like phenotype and assess engraftment and potential therapeutic effects using* in vivo* disease models.

## Supplementary Material

Supplementary Material provided online contains representative images of immunocytochemical stainings for quantitative assessment of expression of skeletal muscle markers (S1) and cardiac markers (S2), relevant positive controls for antibody specificity (S3-S7), qPCR gene expression data for statistical analyses (S8) and representative images from electrophysiological analyses (S9).

## Figures and Tables

**Table 1:** Action potential parameters of cell populations from different cell culture conditions.

	Amplitude (mV)	MDP (mV)	APD (ms)	BF (1/min)	*V*_max_ (V/s)
ISH0	32.1 ± 5.7^§^	−59.6 ± 2.6	234.6 ± 24.9	360.6 ± 81.6	22.6 ± 3.1^§§^
I12	38.3 ± 5.8^##^	−34.2 ± 3.9	85.5 ± 6.8^*∗*^	821.5 ± 92.8^*∗∗∗*^	11.6 ± 1.2^+^
S12	33.1 ± 4.7^#^	−44.1 ± 3.9	106.4 ± 16.2	777.5 ± 73.3^*∗∗*^	17.1 ± 0.7
H12	27.2 ± 2.6	−44.4 ± 3.5	172.7 ± 22.9	444.4 ± 39.3^§§^	15.9 ± 1.7

BF: beating frequency; MDP: maximum diastolic potential; APD: action potential duration; *V*_max⁡_: maximum upstroke velocity. ^*∗*/*∗∗*/*∗∗∗*^ *p* < 0.05/0.01/0.001 (vs. ISH0); ^§/§§/§§§^ *p*  <  0.05/0.01/0.001 (vs. I12); ^+/++/+++^ *p*  <  0.05/0.01/0.001 (vs. S12); ^#/##/###^ *p*  <  0.05/0.01/0.001 (vs. H12).

## Abbreviations

ACTN2: *α*-Actinin 2
AP: Action potential
APD: Action potential duration
CM: Cardiomyocytes
cTnT: Cardiac troponin T
Cx43: Connexin 43
ESC: Embryonic stem cell
GATA4: GATA binding protein 4
ISH0: Fraction of nonadherent cells after preplating
I/S/H: Identifiers for cell culture condition (incubator, shaker, and hanging drop)
MB: Skeletal myoblast
MDP: Maximum diastolic potential
MDSC: Muscle-derived stem cell
MI: Myocardial infarction
Myf3/4: Myogenic regulatory factor 3/4
Nkx2.5: NK2 homeobox 5 protein
Pax7: Paired box gene 7
SCA-1: Stem cell antigen 1
*V*_max_: Maximum AP upstroke velocity.
