# Supplementary material for: Dynamic Support Culture of Murine Skeletal Muscle-Derived Stem Cells Improves Their Cardiogenic Potential In Vitro
Source: Stem Cells Int. 2015 Aug 18;2015:247091. doi: 10.1155/2015/247091 (PMC4556334; doi:10.1155/2015/247091)
Supplement: Supplementary file 1 — Supplementary Material provided online contains representative images of immunocytochemical stainings for quantitative assessment of expression of skeletal muscle markers (S1) and cardiac markers (S2), relevant positive controls for antibody specificity (S3-S7), qPCR gene expression data for statistical analyses (S8) and representative images from electrophysiological analyses (S9). [file 247091.f1.pdf]

Supplementary Figure 1: Skeletal muscle marker expression analysis.

Cells from ISH0 and after twelve days of culture under different conditions were immunocytochemically analyzed for the expression of cardiac markers. Quantification of microscopically assessed expression data is displayed in Figure 3. The panel is divided into cluster staining (upper row) and single cell staining (lower row) pictures. Cells were stained against: Desmin, Pax7, Myf3 and Myf4. Scale: 50 µm.

Supplementary Figure 2: Cardiac muscle marker expression analysis and quantification of non-adherent cells under different culture conditions.

Cells from ISH0 and after twelve days of culture under different conditions were immunocytochemically analyzed for the expression of cardiac markers. Quantification of microscopically assessed expression data is displayed in Figure 3. The panel is divided into cluster staining (upper row) and single cell staining (lower row) pictures. Cells were stained against: ACTN2, cTnT, GATA4 and Nkx2.5. Scale: 50 µm.

Supplementary Figure 3: Antibody specificity control staining

Different control cell types were stained against the indicated markers to verify the specificity of labeling of the target antigen by the primary antibody. The presence of signals in positive control cells and their absence in negative control cells was understood as proper binding of the given antibody. Cross-reactivity was excluded by specific signals in heterogeneous cell populations, e.g. myoblasts including fibroblasts (see Pax7 staining). Cells were stained against: Desmin, Pax7, Myf3 and Myf4, ACTN2, cTnT, Gata4 and Nkx2.5. Scale: 50 µm.

Supplementary Figure 4: Immunocytochemical analysis of ISH0 cells including control staining

Cells from ISH0 were immunocytochemically analyzed for the expression of the indicated markers as previously shown in Supplementary Figure 1 and 2. Shown here in addition are antibody reactivity and background fluorescence and binding control stainings including isotype control plus secondary antibody (Isotype + 2nd Ab) or secondary antibody alone (2nd Ab alone) in contrast to single cell and cluster stainings. Cells were stained against: Desmin, Pax7, Myf3 and Myf4, ACTN2, cTnT, Gata4 and Nkx2.5. Scale: 50 µm.

Supplementary Figure 5: Immunocytochemical analysis of I12 cells including control staining

Cells from I12 were immunocytochemically analyzed for the expression of the indicated markers as previously shown in Supplementary Figure 1 and 2. Shown here in addition are antibody reactivity and background fluorescence and binding control stainings including isotype control plus secondary antibody (Isotype + 2nd Ab) or secondary antibody alone (2nd Ab alone) in contrast to single cell and cluster stainings. Cells were stained against: Desmin, Pax7, Myf3 and Myf4, ACTN2, cTnT, Gata4 and Nkx2.5. Scale: 50 µm.

Supplementary Figure 6: Immunocytochemical analysis of S12 cells including control staining

Cells from S12 were immunocytochemically analyzed for the expression of the indicated markers as previously shown in Supplementary Figure 1 and 2. Shown here in addition are antibody reactivity and background fluorescence and binding control stainings including isotype control plus secondary antibody (Isotype + 2nd Ab) or secondary antibody alone (2nd Ab alone)

in contrast to single cell and cluster stainings. Cells were stained against: Desmin, Pax7, Myf3 and Myf4, ACTN2, cTnT, Gata4 and Nkx2.5. Scale: 50 µm.

Supplementary Figure 7: Immunocytochemical analysis of H12 cells including control staining

Cells from H12 were immunocytochemically analyzed for the expression of the indicated markers as previously shown in Supplementary Figure 1 and 2. Shown here in addition are antibody reactivity and background fluorescence and binding control stainings including isotype control plus secondary antibody (Isotype + 2nd Ab) or secondary antibody alone (2nd Ab alone) in contrast to single cell and cluster stainings. Cells were stained against: Desmin, Pax7, Myf3 and Myf4, ACTN2, cTnT, Gata4 and Nkx2.5. Scale: 50 µm.

Supplementary Figure 8: Expression of cardiomyocyte specific transcripts in MDSC derived cells.

Skeletal myoblasts (MB), purified ESC derived CM and cells from I, S and H cell culture conditions on day 4 and 12 of cultivation were analyzed by qPCR. Expression levels of indicated genes are displayed as ΔCt values relative to the housekeeping control gene β-actin (n=5).

Supplementary Figure 9: Spontaneous activity of MDSCs before conditional culture and under pharmacological stimulation

Spontaneous activity of MDSCs at day 0 measured by current-clamp whole cell patch clamping (**A**+**B**). Left panels in **A** (irregular activity) and **B** (regular activity) show the full trace of a 5 minute measurement. Right panels in **A** and **B** show magnifications of a one second stretch of activity. Spontaneous activity was measured at day 12 under pharmacological stimulation (**C**+**D**). The left panel shows a full 5 second trace before the application of 0.5 mM $CdCl_2$ (**C**) or 1 µM isoproterenol (**D**); the right panel shows a full 5 second trace 30 seconds after application. Scales are labeled accordingly.

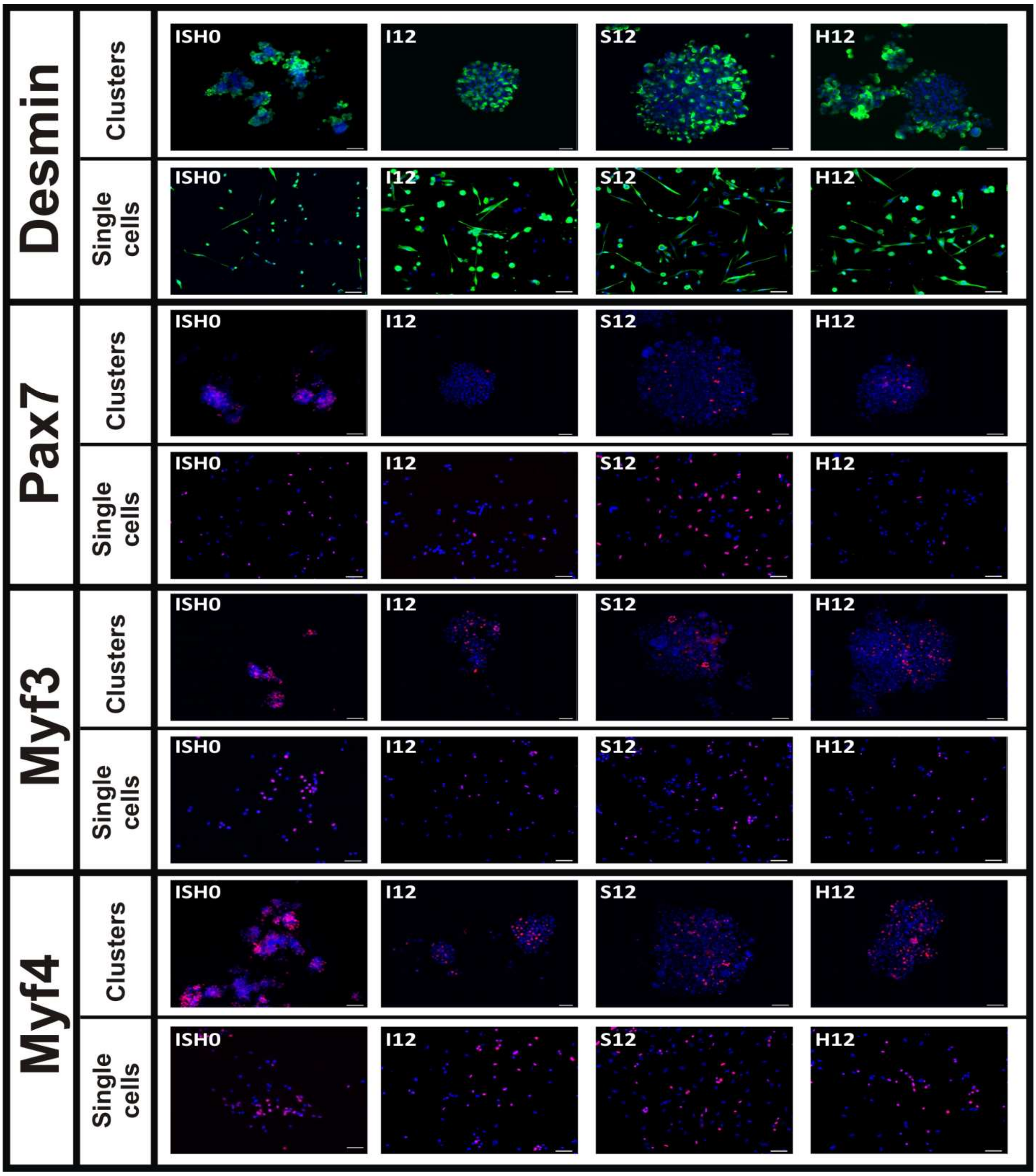

Supplemental Figure 1

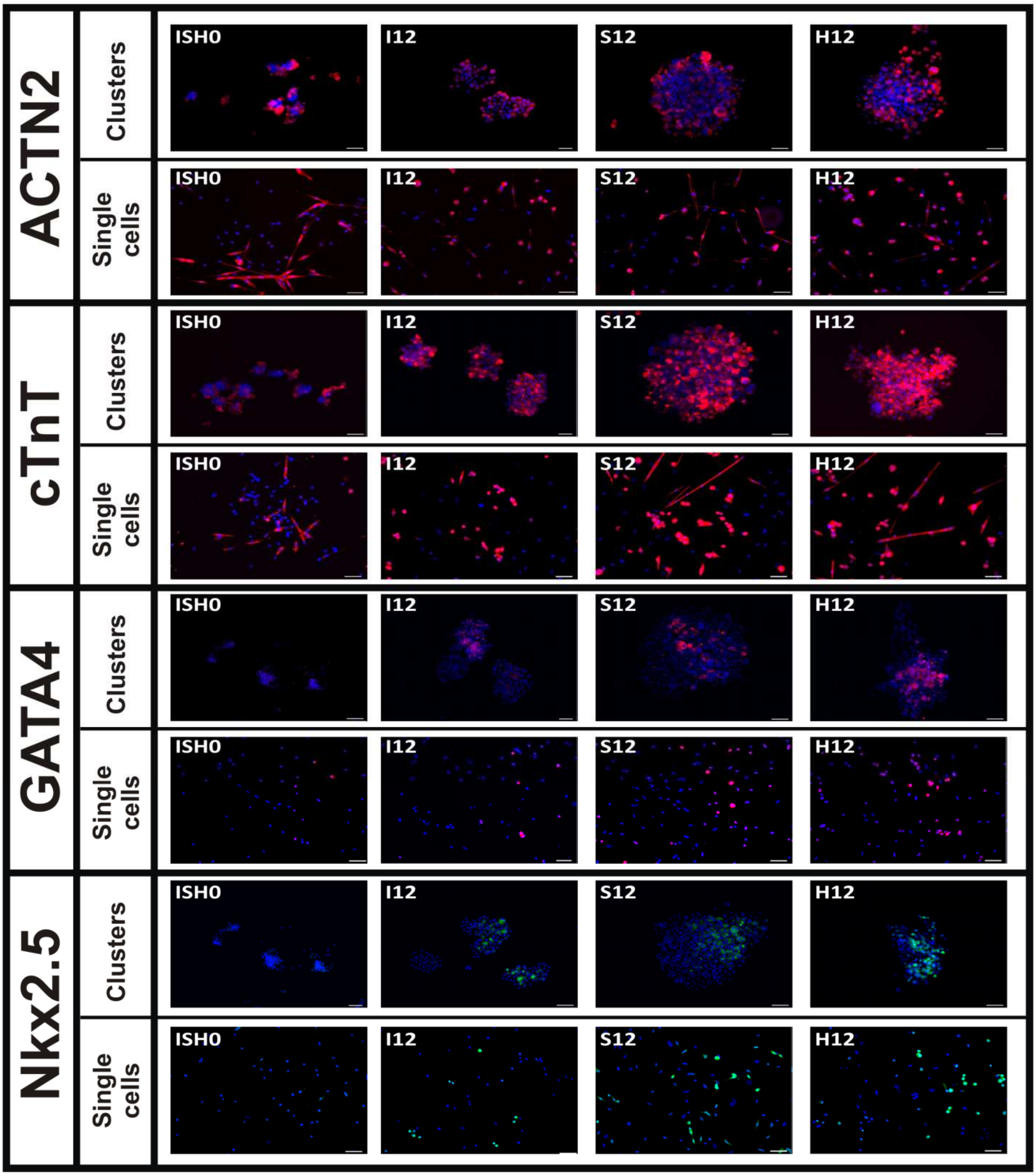

Supplemental Figure 2

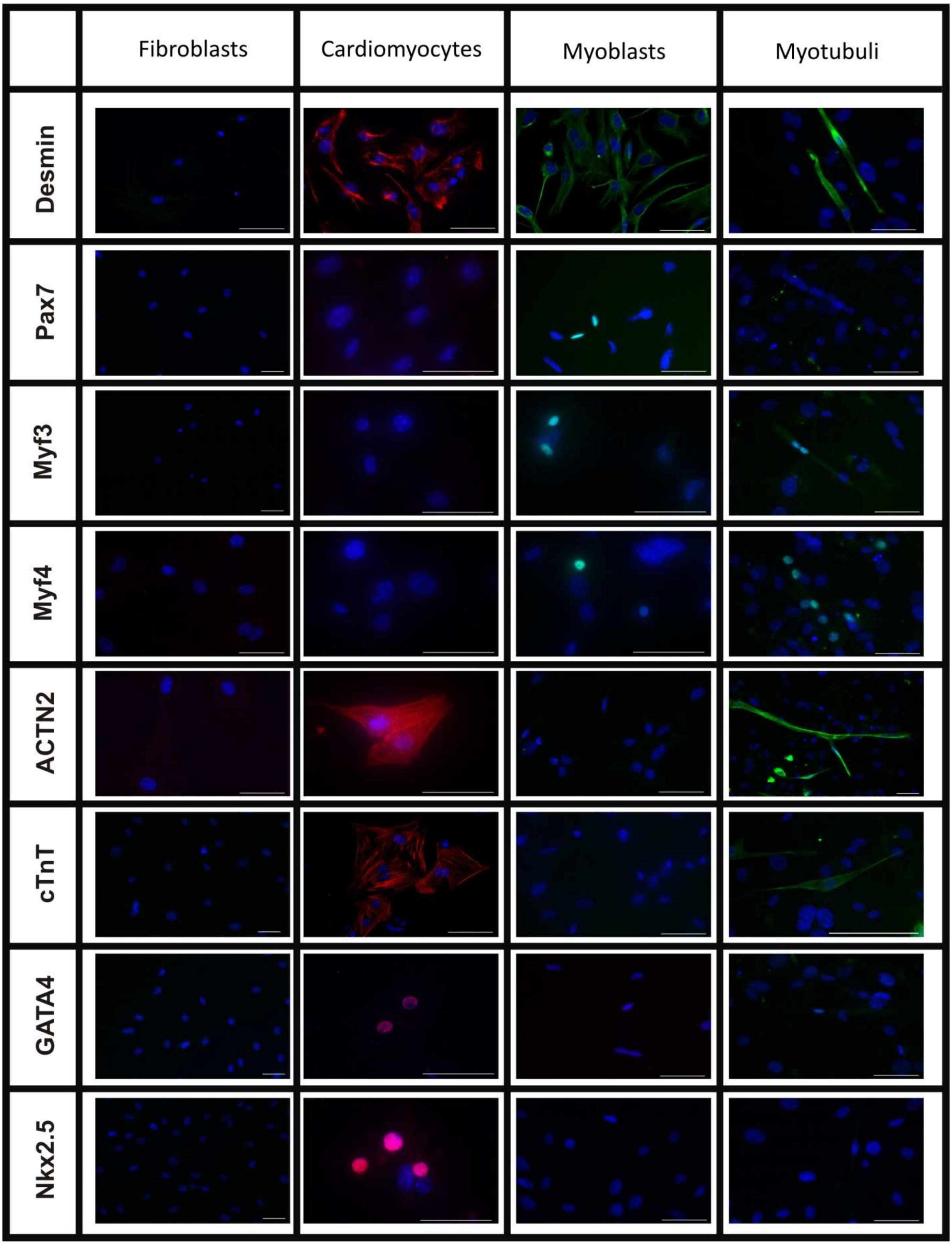

Supplemental Figure 3

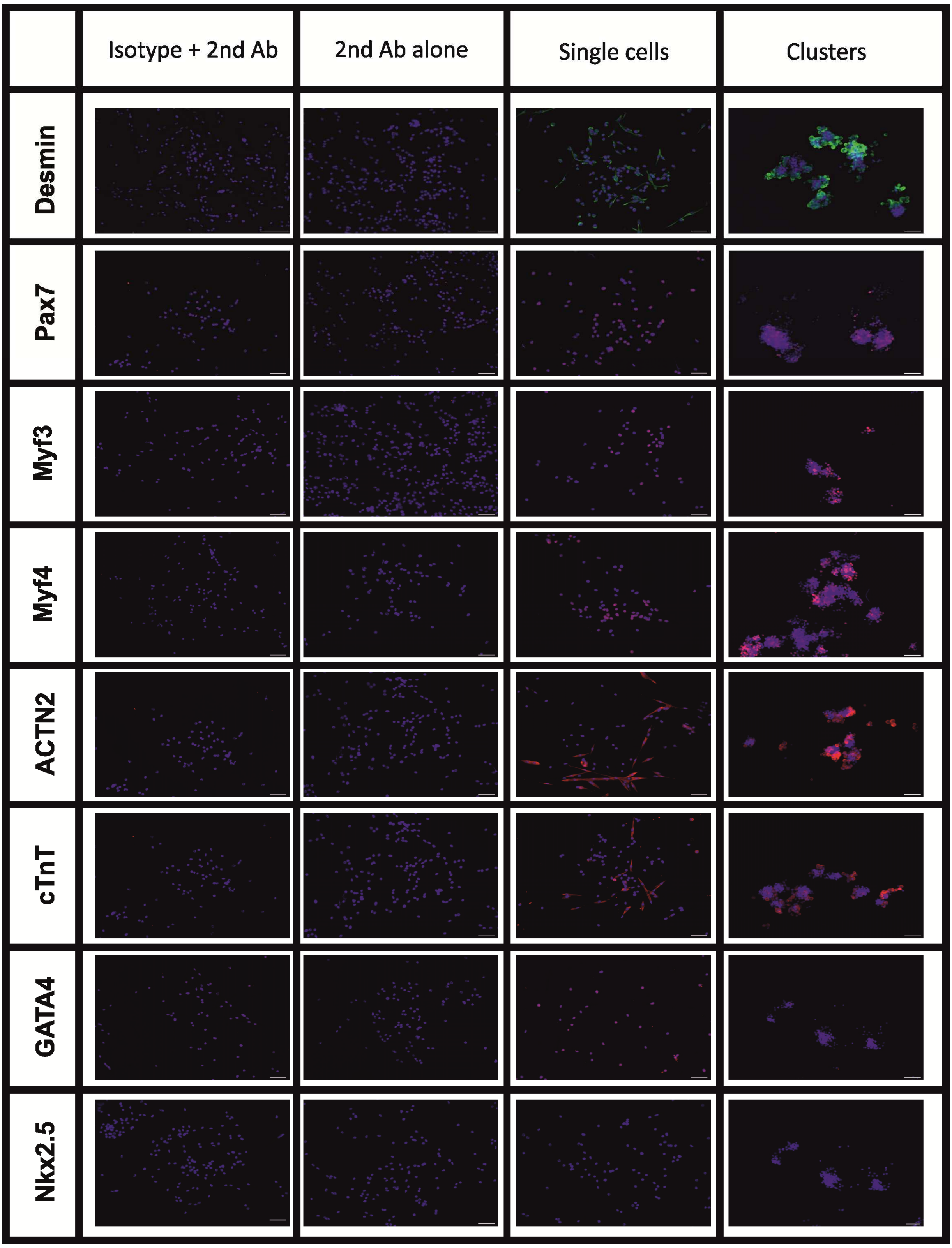

Supplemental Figure 4

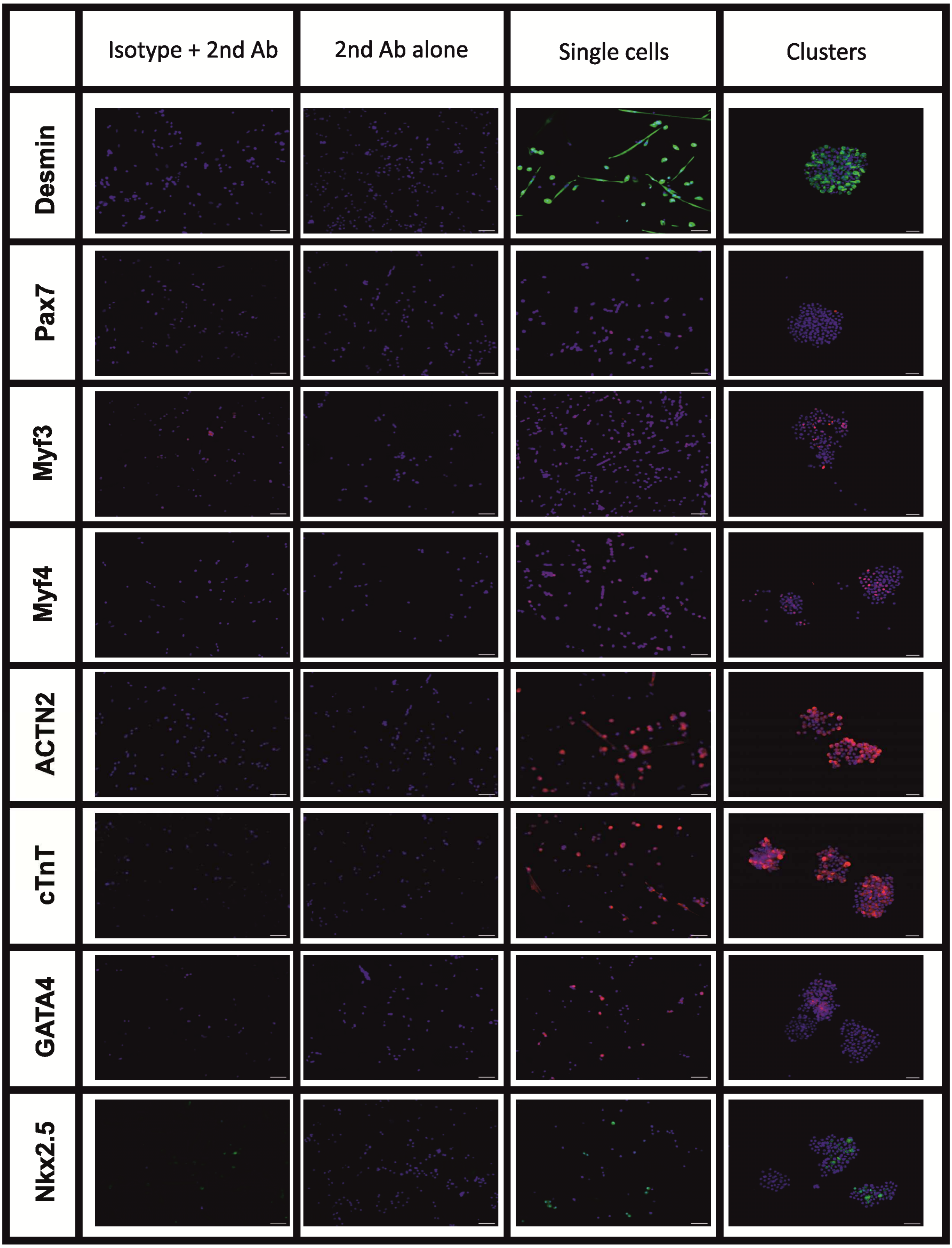

Supplemental Figure 5

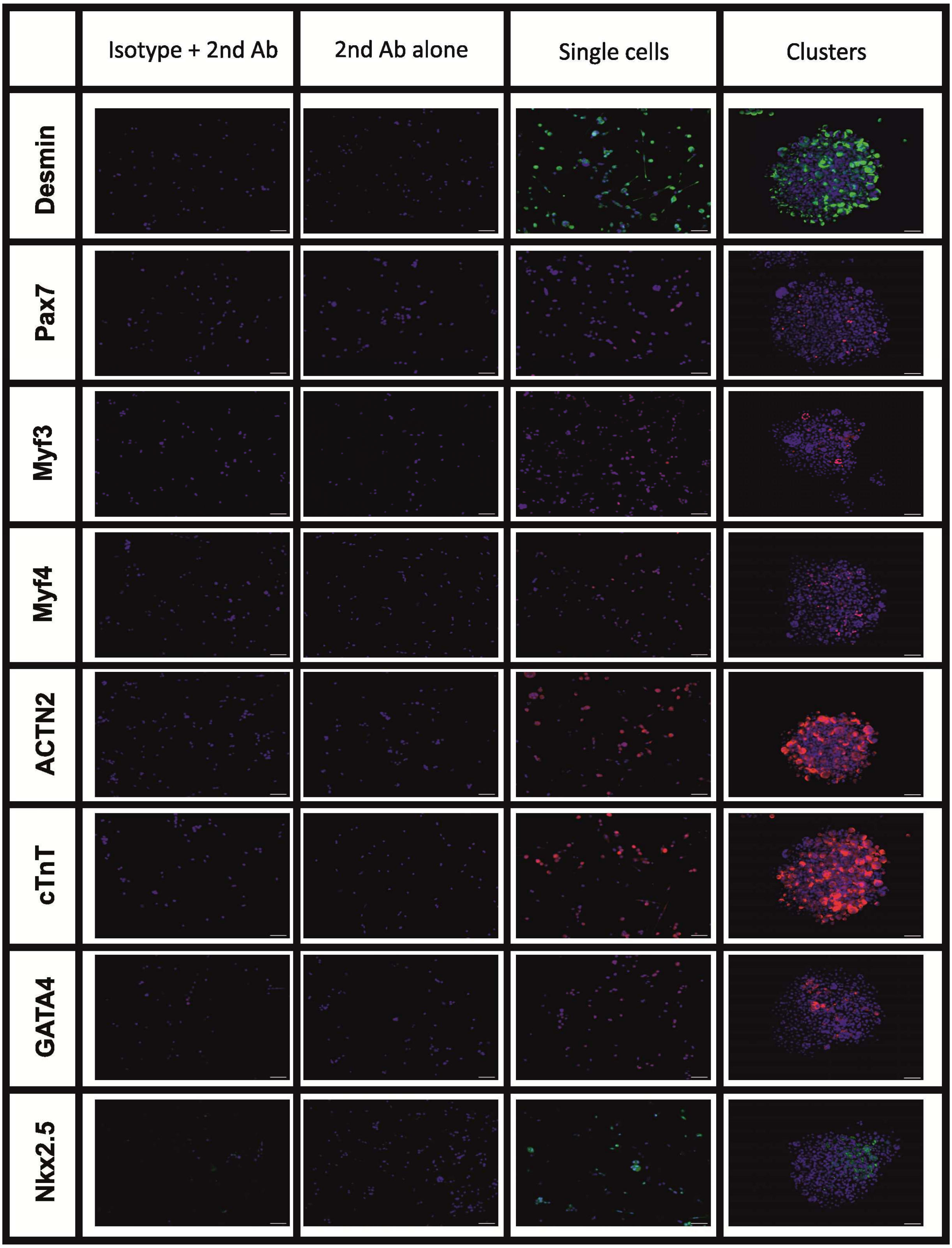

Supplemental Figure 6

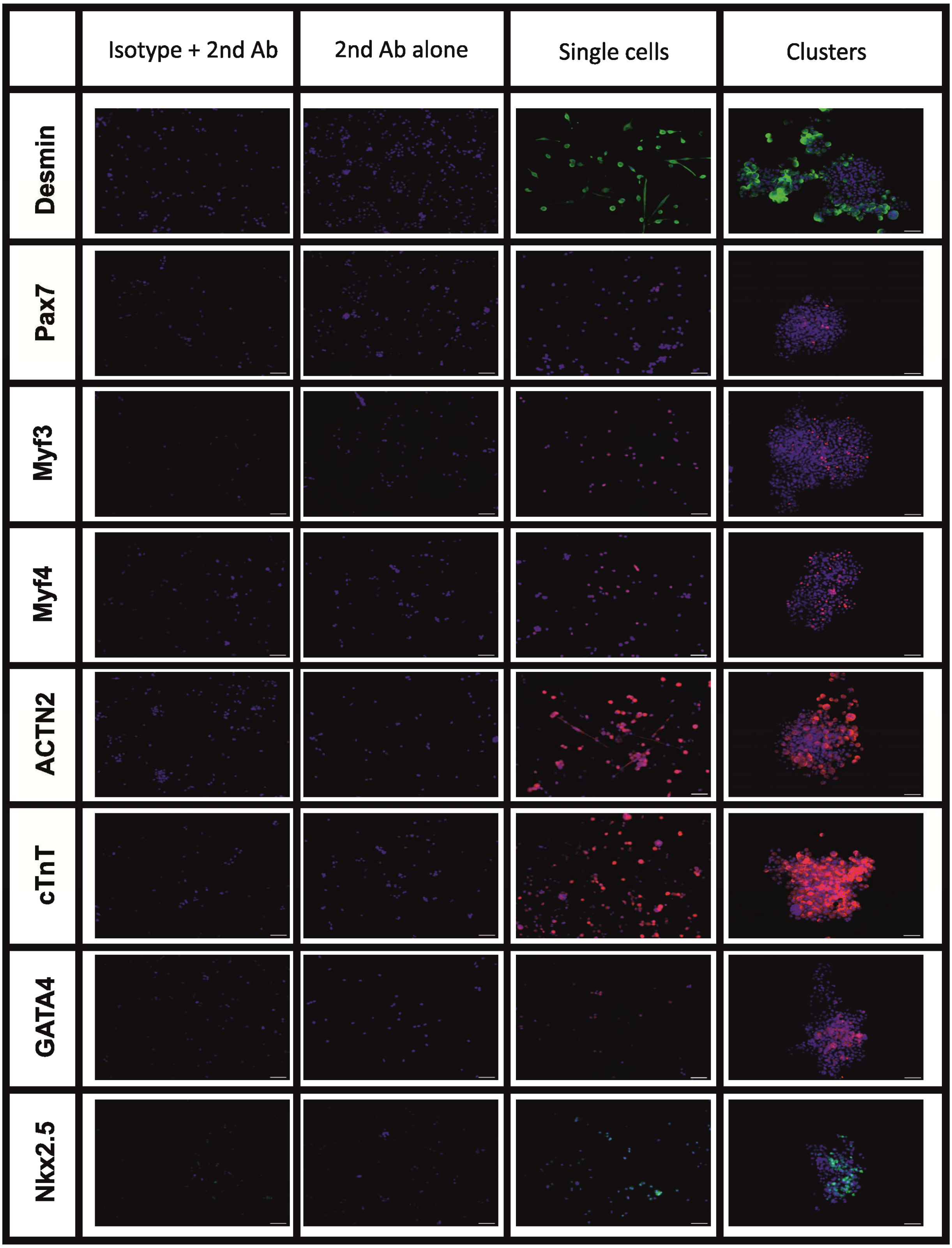

Supplemental Figure 7

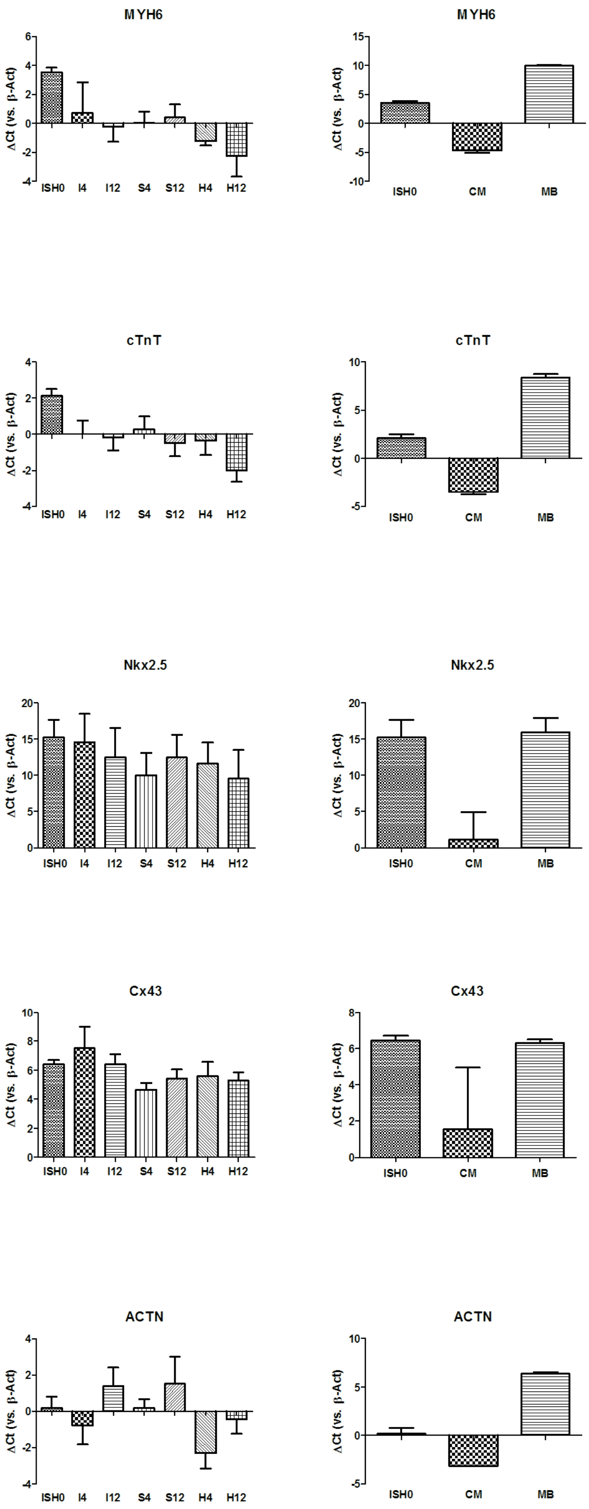

Supplemental Figure 8

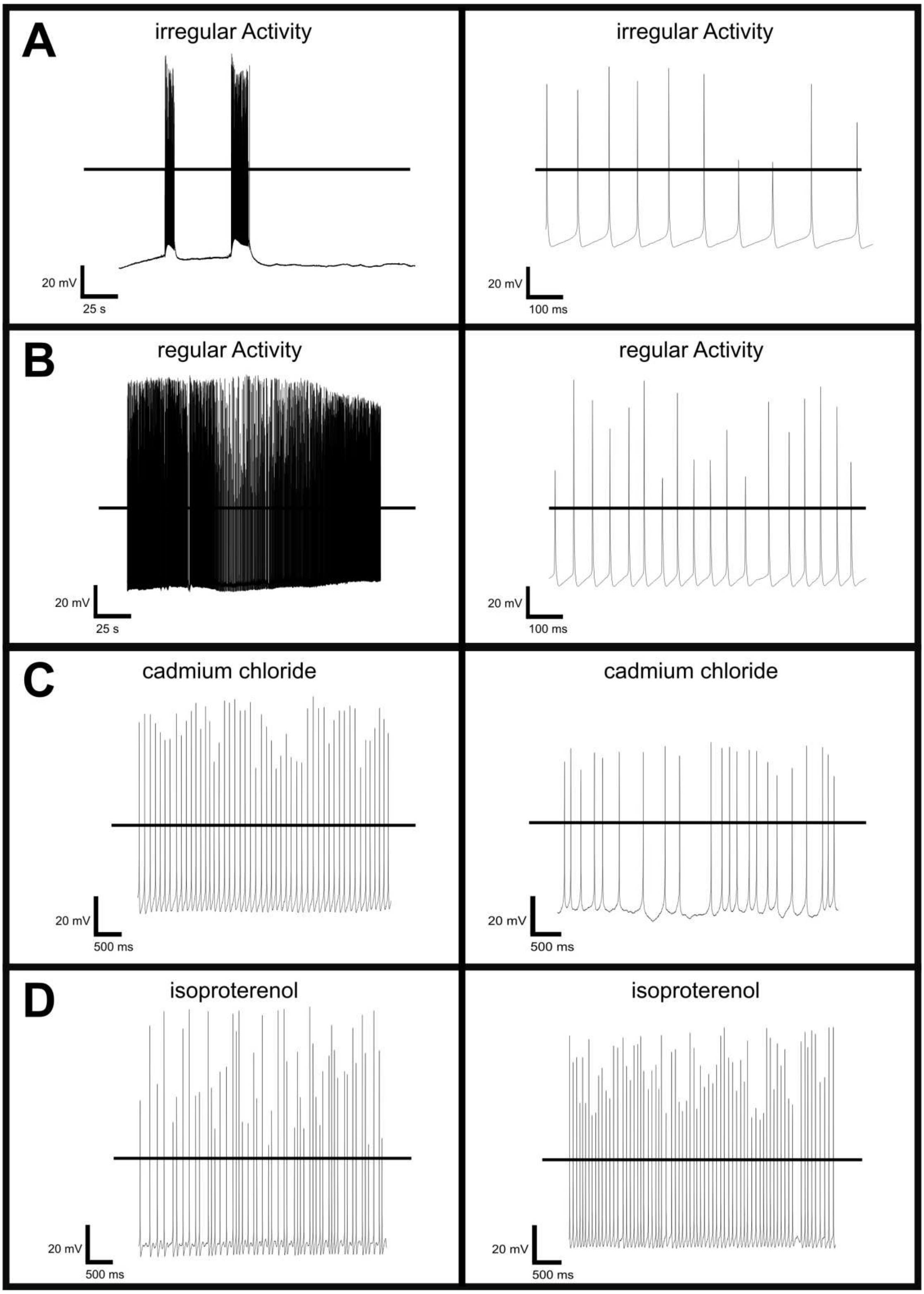

Supplemental Figure 9

Supplementary Table 1: Primary and secondary antibodies used for immunological staining and flow cytometry.

Antibody	Company	Conjugation	Species	Reactivity	Dilution
Desmin	Gene Tex (GTX15200)	x	Rabbit	Mouse	1:200
cTnT	Santa Cruz (sc-20025)	x	Mouse	Mouse	1:200
Gata4	Santa Cruz (sc-25310)	x	Mouse	Mouse	1:200
Pax7	Santa Cruz (sc-81648)	x	Mouse	Mouse	1:200
Nkx2.5	Santa Cruz (sc-14033)	x	Rabbit	Mouse	1:200
α-ACT	Sigma (A7811)	x	Mouse	Mouse	1:500
Myf3	Abcam (ab16148)	x	Mouse	Mouse	1:200
Myf4	Abcam (ab1835)	x	Mouse	Mouse	1:200
Sca-1	BD (553336)	PE	Rat	Mouse	1:800
CD34	Biolegend (119307)	FITC	Rat	Mouse	1:400
IgG-Isotype	Santa Cruz (sc-2025)	x	Mouse	x	1:100
IgG-Isotype	Santa Cruz (sc-2027)	x	Rabbit	x	1:100
IgG2a-Isotype	BD (553930)	PE	Rat	x	1:200
IgG2a-Isotype	Biolegend (400507)	FITC	Rat	x	1:200
Alexa Fluor® 488	Invitrogen (A11001)	AF488	Goat	Mouse	1:1000
Alexa Fluor® 488	Invitrogen (A11008)	AF488	Goat	Rabbit	1:1000
Alexa Fluor® 568	Invitrogen (A11004)	AF568	Goat	Mouse	1:1000
Alexa Fluor® 568	Invitrogen (A11011)	AF568	Goat	Rabbit	1:1000
DAPI	Invitrogen (D3571)	x	x	x	1:1000

Supplementary Table 2: Primers used for quantitative RT-PCR.

Gene	NCBI Reference-Sequence	forward / reverse	Sequence
b-Act	NM_007393.3	F	AGGTCATCACTATTGGCAACGA
		R	CAACGTCACACTTCATGATGGA
Nkx2.5	NM_008700.2	F	CACCGCGTCGCCACCATGTTCCCCA
		R	CGGGGCGCCCCAAACGGGCT
MHC6	NM_001164171.1	F	GCTGGAAGATGAGTGCTCAGAG
		R	CCAGCCATCTCCTCTGTTAGGT
ACTN2	NM_134156.2	F	TCGCCAAGTGTCAACGCTCGTT
		R	GGTCGATGGTTTCCAGCAGCTT
Cx43	NM_010288.3	F	CTCCTCCTGGGTACAAGCTG
		R	TAAGGGCTGGAGTTCGTGTC
cTnT	NM_011619.2	F	GCTACAGACTCTGATCGAGGCT
		R	GCTCATTGCGAATACGCTGCTG

Supplementary Table 3 A: Composition of patch clamp pipette solutions.

Type	Reagent	Company	Concentration [mM]
**Sodium / Calcium**	NaCl	Roth	120
	$MgCl_2*6H_2O$	Merck	1
	KCl	Merck	5
	TEA-Cl	Sigma	20
	$CaCl_2*2H_2O$	Merck	3.6
	Hepes	Sigma	10
	TEA-OH	Sigma	ad. pH 7.4
**Potassium**	NMG-Cl	Sigma	135
	$MgCl_2*6H_2O$	Merck	1
	KCl	Merck	5
	$CaCl_2*2H_2O$	Merck	3.6
	$NiCl_2$	Merck	3
	Hepes	Sigma	10
	NMG	Sigma	ad. pH 7.4
**Action Potentials**	NaCl	Roth	140
	$MgCl_2*6H_2O$	Merck	1
	KCl	Merck	5.4
	$CaCl_2*2H_2O$	Merck	1.8
	D-Glucose	Sigma	5
	Hepes	Sigma	10
	NaOH	Sigma	ad. pH 7.4

Supplementary Table 3 B: Composition of external patch clamp solutions.

Type	Reagent	Company	Concentration [mM]
**Sodium / Calcium**	CsCl	Fluka	120
	$MgCl_2*6H_2O$	Merck	3
	MgATP	Sigma	5
	EGTA	Fluka	10
	Hepes	Sigma	5
	CsOH	Sigma	ad. pH 7.4
**Action Potentials / Potassium**	MgCl2*6H2O	Merck	1
	KCl	Merck	50
	Hepes	Sigma	10
	EGTA	Fluka	10
	MgATP	Sigma	3
	K-Aspartate	Sigma	80
	KOH	Fluka	ad. pH 7.4
